# Supplementary material for: Comparison of the Oxidative Stability and Antioxidant Activity of Extra-Virgin Olive Oil and Oils Extracted from Seeds of Colliguaya integerrima and Cynara cardunculus under Normal Conditions and After Thermal Treatment
Source: Antioxidants (Basel). 2019 Oct 9;8(10):470. doi: 10.3390/antiox8100470 (PMC6827052; doi:10.3390/antiox8100470)
Supplement: Supplementary file 1 [file antioxidants-08-00470-s001.pdf]

Supporting Information for:

Comparison of the Oxidative Stability and Antioxidant Activity of Extra-Virgin Olive Oil and Oils Extracted from Seeds of *Colliguayaintegerrima and Cynara cardunculus* under Normal Conditions and After Thermal Treatment.

Diana Abril[a]. Yaneris Mirabal-Gallardo[b]. AymeéGonzález[a]. Adolfo Marican[c].Esteban F. Durán-Lara[d]. Leonardo S. Santos[e]. Oscar Valdés[b*]

[a]Departamento de Biología y Química. Facultad de CienciasBásicas. Universidad Católica del Maule. Talca. Chile.

[b]Vicerrectoría de Investigación y Postgrado. Universidad Católica del Maule. Talca. Chile.

[c]Chemistry Institute of Natural Resources. University of Talca. P.O. Box 747. Talca. Chile.

[d]Biomaterials Laboratory. Drug Delivery and Controlled Release. Departamento de Microbiología. Facultad de Ciencias de la Salud. Universidad de Talca. Talca

[e]Laboratory of Asymmetric Synthesis. Chemistry Institute of Natural Resources. University of Talca. P.O. Box 747. Talca. Chile.

* Corresponding Authors: Oscar Valdés
Address: Vicerrectoría de Investigación y Postgrado. Universidad Católica del Maule. Talca. Chile.
Email address: ovaldes@ucm.cl | Phone: +56(71) 2203304

Table S1. GC-MS analysis of *Colliguaya integerrima* oil before and after heated at 180 ºC. The results were expressed as mean values ± SDs (n=3). Same letter beside standard devision (SD) in the same row indicate no statistical differences between the oils, using Tukey HSD (at 95% level of confidence).

Compound Detected	Retention Time (min)	% Area (Heat)	% Area (Cold)
**Tetradenoic acid**	15.96	0.05 ± 0.01a	0.05 ± 0.01a
**Pentadecanoic acid**	16.76	0.010 ± 0.006a	0.010 ± 0.004a
**Hexadecanoic acid. methyl ester**	17.26	0.42 ± 0.04a	0.53 ± 0.02b
**n-Hexadecanoic acid**	17.67	4.30 ± 0.35a	6.31 ± 0.25b
**9,12-Octadecadienoic acid (Z.Z)-. methyl ester**	18.48	0.61 ± 0.07a	0.64 ± 0.15a
**9-Octadecenoic acid. methyl ester (E)-**	18.50	0.12 ± 0.06a	0.15 ± 0.05a
**9,12,15-Octadecatrienoic acid. methyl ester. (Z.Z.Z)-**	18.52	0.16 ± 0.012a	0.18 ± 0.010a
**Methyl stearate**	18.63	0.05 ± 0.013a	0.12 ± 0.07a
**9,12-Octadecadienoic acid (Z.Z)-**	18.95	20.23 ± 1.16a	20.70 ± 1.11a
**cis-11-Eicosenoic acid. methyl ester**	19.73	0.50 ± 0.11a	0.51 ± 0.17a

cis-13- Eicosenoic acid	20.05	1.80 ± 0.15a	2.40 ± 0.09b
9.12-Octadecadienoic acid (Z.Z)-. 2.3-dihydroxypropyl ester	20.45	0.11 ± 0.05a	0.14 ± 0.04a
9.12.15-Octadecatrienoic acid. 2.3-dihydroxypropyl ester. (Z.Z.Z)-	20.71	0.91 ± 0.16a	1.31 ± 0.10b
Glycerol 1-palmitate	20.91	0.10 ± 0.010a	0.12 ± 0.013a
Linoleic acid ethyl ester	21.62	0.004 ± 0.002a	0.005 ± 0.001a
9.12-Octadecadienoic acid (Z.Z)-. 2.3-dihydroxypropyl ester	21.85	0.93 ± 0.06a	0.95 ± 0.07a
Linolenic acid. 2-hydroxy-1-(hydroxymethyl) ethyl ester (Z.Z.Z)-	21.91	0.22 ± 0.04a	0.49 ± 0.02b
Tetracosanoic acid. methyl ester	21.95	0.14 ± 0.06a	0.18 ± 0.08a
Squalene	22.49	0.09 ± 0.02a	0.11 ± 0.02a
γ-Tocopherol	23.97	0.27 ± 0.02a	0.41 ± 0.01b
Vitamin E	24.61	0.15 ± 0.03a	0.22 ± 0.02b
Olean-13(18)-ene	24.98	0.15 ± 0.05a	0.20 ± 0.06a
Campesterol	25.68	0.05 ± 0.02a	0.06 ± 0.01a
Stigmasterol	25.98	0.05 ± 0.01a	0.06 ± 0.01a
γ-Sitosterol	26.65	1.08 ± 0.15a	1.34 ± 0.13a

Table S2. GC-MS analysis of *Cynara cardunculus* oil before and after being heated at 180 ºC. The results were expressed as mean values ± SDs (n=3). Same letter beside SD in the same row indicate no statistical differences between the oils, using Tukey HSD (at 95% level of confidence).

	Retention Time (min)	% Area (Heat)	% Area (Cold)
Tetradenoic acid	15.96	0.16 ± 0.02a	0.20 ± 0.03a
Pentadecanoic acid	16.76	0.03 ± 0.01a	0.04 ± 0.01a
Hexadecanoic acid. methyl ester	17.26	0.65 ± 0.04a	0.75 ± 0.02b
n-Hexadecanoic acid	17.73	8.37 ± 0.15a	9.06 ± 0.11b
9,12-Octadecadienoic acid (Z.Z)-. methyl ester	18.48	2.17 ± 0.29a	2.43 ± 0.36a
9 Octadecenoic acid. methyl ester (E)-	18.50	0.66 ± 0.09a	0.72 ± 0.14a
Methyl stearate	18.63	0.22 ± 0.03a	0.23 ± 0.02b
9.12-Octadecadienoic acid (Z.Z)-	18.95	28.88 ± 1.63a	29.97 ± 1.43a
cis-11-Eicosenoic acid. methyl ester	19.73	0.14 ± 0.01a	0.15 ± 0.08a
9.12-Octadecadienoic acid (Z.Z)-. 2.3-dihydroxypropyl ester	20.45	0.08 ± 0.01a	0.18 ± 0.01b
9.12.15-Octadecatrienoic acid. 2.3-dihydroxypropyl ester. (Z.Z.Z)-	20.71	0.95 ± 0.06a	1.21 ± 0.09b
Glycerol 1-palmitate	20.91	0.16 ± 0.08a	0.18 ± 0.06a
Linoleic acid ethyl ester	21.62	0.11 ± 0.07a	0.12 ± 0.08a

**9.12-Octadecadienoic acid (Z.Z)-. 2.3-dihydroxypropyl ester**	21.85	1.84 ± 0.39a	3.06 ± 0.15b
**Tetracosanoic acid. methyl ester**	21.95	0.13 ± 0.06a	0.14 ± 0.04a
**Squalene**	22.49	0.03 ± 0.01a	0.04 ± 0.01a
**Vitamin E**	24.61	0.79 ± 0.06a	1.07 ± 0.05b
**4-[[2-(3.4-dimethoxyphenyl) ethylamino]methyl]-2-methoxy-. Phenol.**	25.11	0.13 ± 0.01a	0.27 ± 0.07b
**Campesterol**	25.68	0.11 ± 0.04	0.11 ± 0.08
**Stigmasterol**	25.98	0.10 ± 0.06a	0.13 ± 0.05a
**γ-Sitosterol**	26.65	0.26 ± 0.11a	0.32 ± 0.14a
**á-Amyrin**	27.19	0.09 ± 0.01a	0.10 ± 0.01a
**Stigmast-7-en-3-ol. (3á.5á)-**	27.30	0.10 ± 0.08a	0.14 ± 0.03a
**à-Amyrin**	27.80	0.34 ± 0.01a	0.36 ± 0.02a
